# Clinical Effects of Oblique Lateral Interbody Fusion by Conventional Open *versus* Percutaneous Robot‐Assisted Minimally Invasive Pedicle Screw Placement in Elderly Patients

**DOI:** 10.1111/os.12587

**Published:** 2019-12-27

**Authors:** Shuo Feng, Wei Tian, Yi Wei

**Affiliations:** ^1^ Department of Spine Surgery Beijing Jishuitan Hospital Beijing China

**Keywords:** Minimally invasive, Oblique lumbar interbody fusion, Pedicle screw, Percutaneous, Robot

## Abstract

**Objectives:**

To compare the clinical outcomes of percutaneous robot‐assisted minimally invasive pedicle screw insertion *versus* freehand fluoroscopy‐assisted pedicle screw insertion using a traditional open technique in elderly patients undergoing an oblique lumbar interbody fusion (OLIF) procedure.

**Methods:**

Based on the inclusion and exclusion criteria, 80 patients with lumbar degenerative disease who attended the spinal surgery department of the Beijing Jishuitan Hospital between January 2017 and April 2018 were enrolled in the present study. Patients were randomized 1:1 to undergo percutaneous robot‐assisted minimally invasive pedicle screw insertion (experimental group, *n* = 40) or freehand fluoroscopy‐assisted pedicle screw insertion using a traditional open technique (control group, *n* = 40). Outcomes were accuracy of screw placement evaluated on postoperative CT using the modified Gertzbein and Robbins scale, operative time, blood loss, postoperative drainage, lower back and leg pain evaluated using a visual analogue scale (VAS), lumbar function evaluated using the Oswestry disability index (ODI), and complication rates.

**Results:**

A total of 344 vertebral pedicle screws were inserted: 170 screws in the experimental group, and 174 screws in the control group. Accurate screw placement was significantly higher in the experimental group (98.2% [167/170]) than in the control group (93.1% [162/174]). Clinical outcomes showed significant differences between the experimental and control groups in operative time, intraoperative blood loss, and postoperative VAS for lower back pain in the immediate postoperative period.

**Conclusion:**

Robot‐assisted pedicle screw insertion in OLIF is an effective strategy for the management of elderly patients with lumbar degenerative diseases.

## Introduction

Population aging has become a challenge to healthcare systems globally. In particular, the incidence of spinal degenerative disease is rising. Surgical treatment of spinal degenerative disease usually involves lumbar intervertebral fusion and pedicle screw internal fixation. However, this approach may be challenging in elderly patients, especially in those with severe orthopaedic degeneration and osteoporotic vertebrae, as pedicle screw insertion may breach the cortical wall, which can cause nerve damage, decrease the holding strength of the screw, and increase the risk of bone cement leakage^1^, ^2^. In addition, surgery in elderly patients can be associated with complications due to the presence of comorbidities and poor cardiopulmonary function. Consequently, there remains an unmet need to minimize surgical risk in elderly patients undergoing surgery for the treatment of spinal degenerative disease by improving precision in surgery, reducing surgical trauma, shortening operative times, and decreasing intraoperative hemorrhage.

Evidence suggests that the safety of fixation in spinal degenerative disease is enhanced by controlling the trajectory of the pedicle screw to obtain accurate screw placement without compromising the integrity of the cortical bone^3^, ^4^. In 2012, Silvestre was the first to report on oblique lumbar interbody fusion (OLIF) as a minimally invasive technique for spinal fusion^5^. OLIF involves an oblique anterior approach for intervertebral fusion and a posterior approach for pedicle screw fixation. An intervertebral cage is placed between the left psoas major muscle and the abdominal aorta, *via* an oblique anterior approach, which reduces bone destruction and blood loss compared to traditional posterior lumbar interbody fusion. However, posterior fixation using freehand fluoroscopy‐assisted pedicle screw insertion with an open technique is associated with trauma and complications during and after surgery.

**Figure 1:**
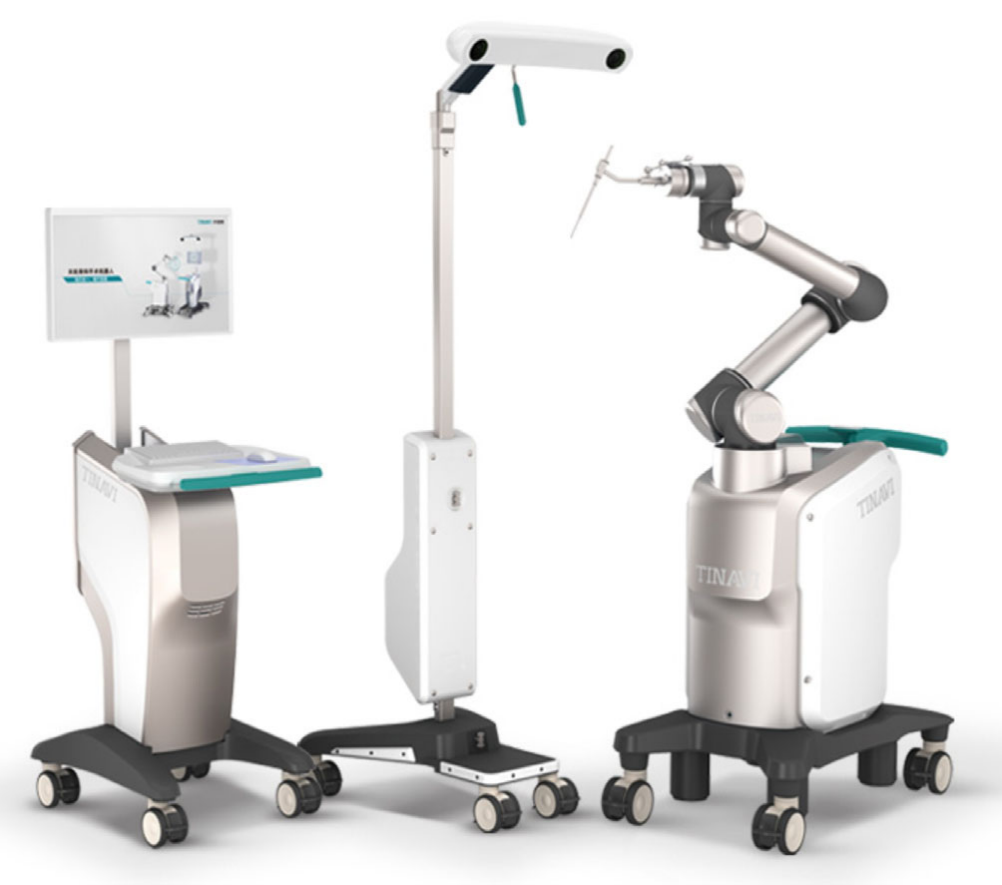
The “TiRobot” orthopaedic robot, developed by TINAVI Medical Technologies, provides a new technology that facilitates the precise placement of pedicle screws. Main components of the TiRobot: robot arm, optical tracking device, surgical planning, and control workstation.

New technologies offer clinicians additional options. “TiRobot” (Fig. 1), an orthopaedic robot developed by Beijing TINAVI Medical Technologies, provides a new technology for minimally invasive, percutaneous pedicle screw placement. This technique has the potential to shorten operative time, reduce intraoperative blood loss, improve the accuracy of fixation, and reduce surgical risk in elderly patients.

In this study, we compared the clinical outcomes of percutaneous robot‐assisted minimally invasive pedicle screw insertion versus freehand fluoroscopy‐assisted pedicle screw insertion in elderly patients undergoing an OLIF procedure. Our research questions included the following. First, is percutaneous robot‐assisted minimally invasive pedicle screw insertion in patients undergoing an OLIF procedure feasible and safe? Second, does percutaneous robot‐assisted minimally invasive pedicle screw insertion result in a stable internal fixation, reduced surgical trauma, and early postoperative recovery in elderly patients with lumbar degenerative diseases? Third, what perioperative complications are associated with percutaneous robot‐assisted minimally invasive pedicle screw insertion?

## Methods

### *Study Population*

Inclusion criteria followed the P, participant; I, intervention; C, comparison; O, outcome; S, study design principle: (i) Participant (P): patients aged ≥55 years with lumbar degenerative disease who were undergoing an OLIF procedure; (ii) Intervention (I): percutaneous robot‐assisted minimally invasive pedicle screw insertion; (iii) Comparison (C): freehand fluoroscopy‐assisted pedicle screw insertion by a traditional open technique; (iv) Outcome (O): accuracy of screw placement, operative time, blood loss, postoperative drainage, lower back and leg pain evaluated using a visual analogue scale (VAS), lumbar function evaluated using the Oswestry disability index (ODI), and complication rates; and (v) study design (S): randomized controlled trial. Exclusion criteria were: (i) age <55 years; (ii) presence of systemic infection or local infection in the target surgical site; (iii) presence of malignant tumor; or (iv) patients who refused surgery. Patients were randomized 1:1 to undergo percutaneous robot‐assisted minimally invasive pedicle screw insertion (experimental group) or freehand fluoroscopy‐assisted pedicle screw insertion using a traditional open technique (control group). All patients underwent surgery performed by the same team of experienced surgeons.

### *Surgical Procedure*

Preoperative imaging using X‐ray, CT, and MRI was performed in all patients to confirm diagnosis and surgical indication. All patients underwent a two‐step surgical procedure that included OLIF and posterior fixation with pedicle screws.

**Figure 2:**
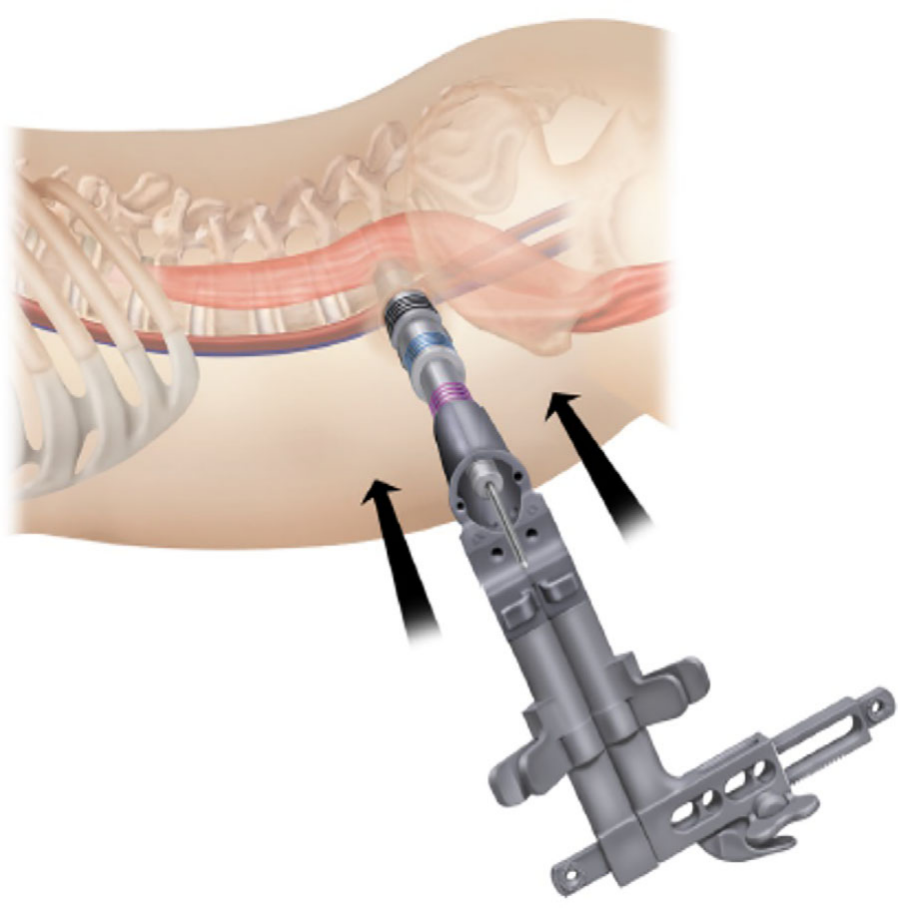
With the guide wire or first dilator in place and firmly impacted into the annulus, sequential dilation was used to spread the fibers of the abdominal musculature. The retractor assembly was attached to the flexible arm to maintain the retractor position.

**Figure 3:**
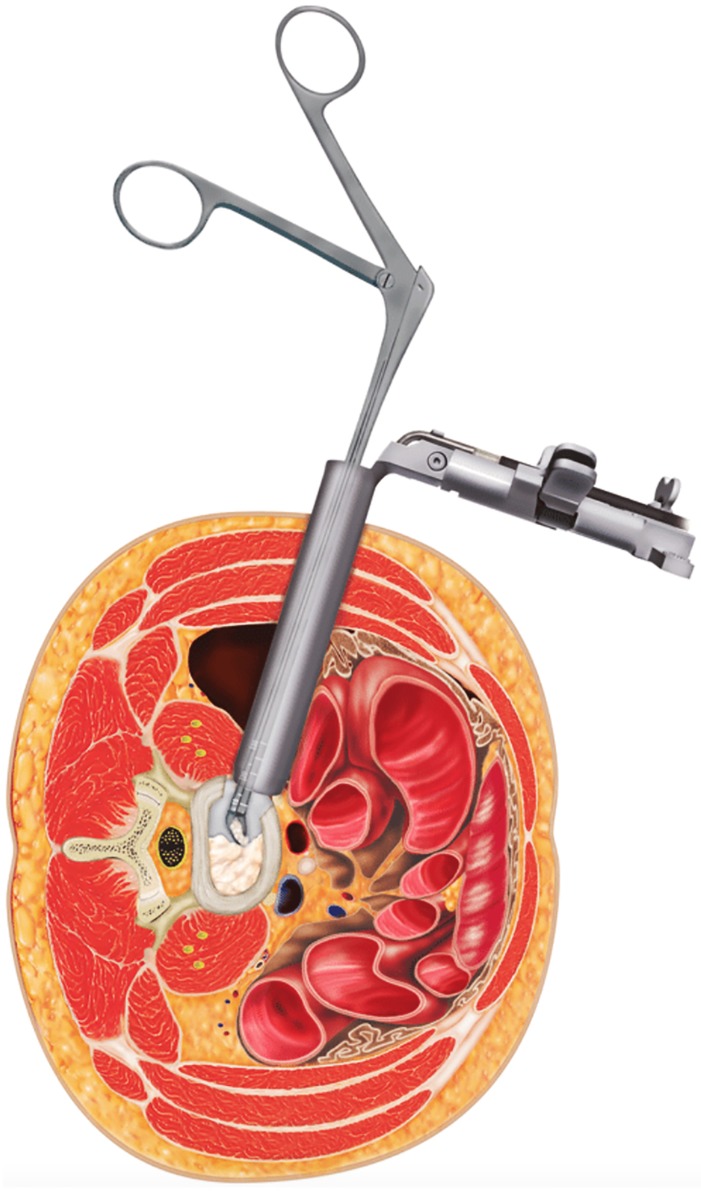
The nucleus pulposus of the intervertebral disc and the cartilage endplate were removed using a pituitary rongeur and other disc preparation instruments. It is extremely important that the end plates are meticulously prepared for fusion by removing the cartilaginous disc without destroying the cortical end plates.

**Figure 4:**
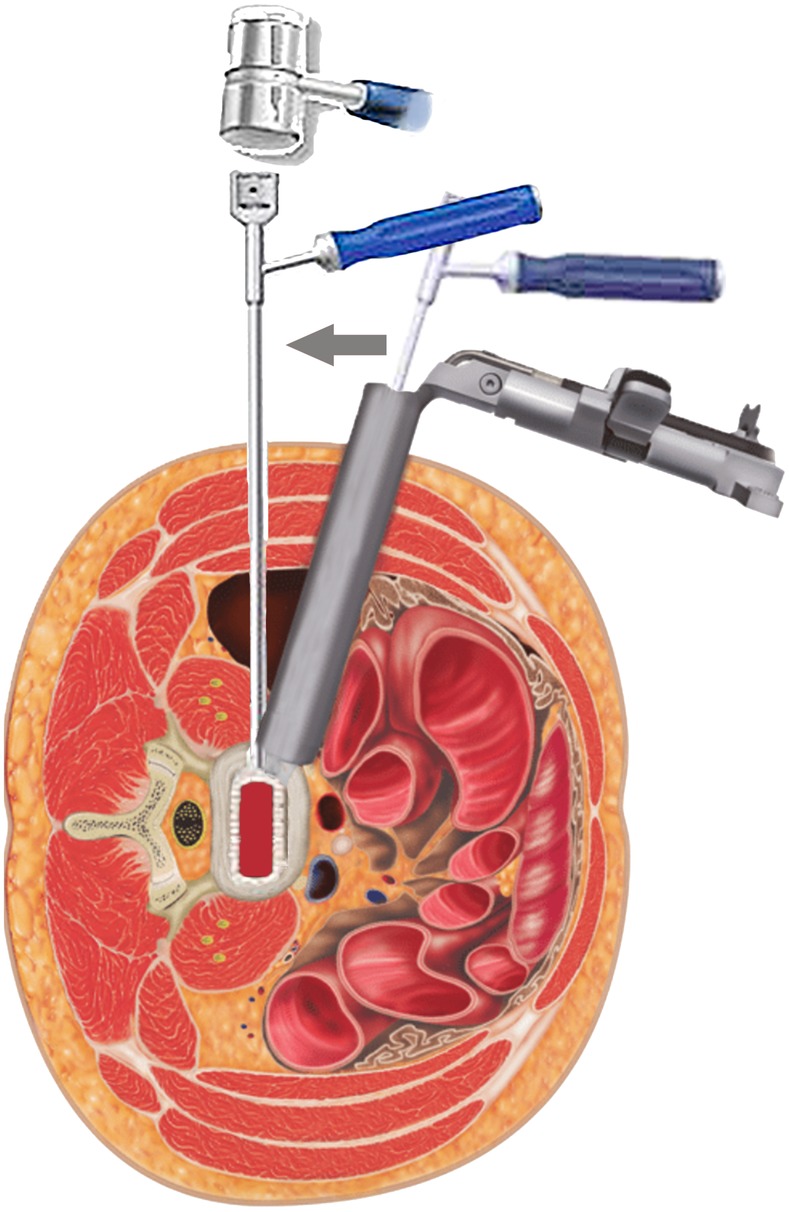
An intervertebral fusion cage was inserted. A mallet was used to gently insert the fusion cage while monitoring placement under AP fluoroscopy. The inserter entered obliquely and was turned to allow the surgeon to place it orthogonally across the disc space.

First, patients were placed in the right lateral position, and the target segment was located with fluoroscopy. An incision was made, a K‐wire was inserted into the intervertebral space, and a dilator was introduced over the K‐wire (Fig. 2). The nucleus pulposus of the intervertebral disc and the cartilage endplate were removed (Fig. 3), and an intervertebral fusion cage was inserted (Fig. 4)^6^, ^7^.

**Figure 5:**
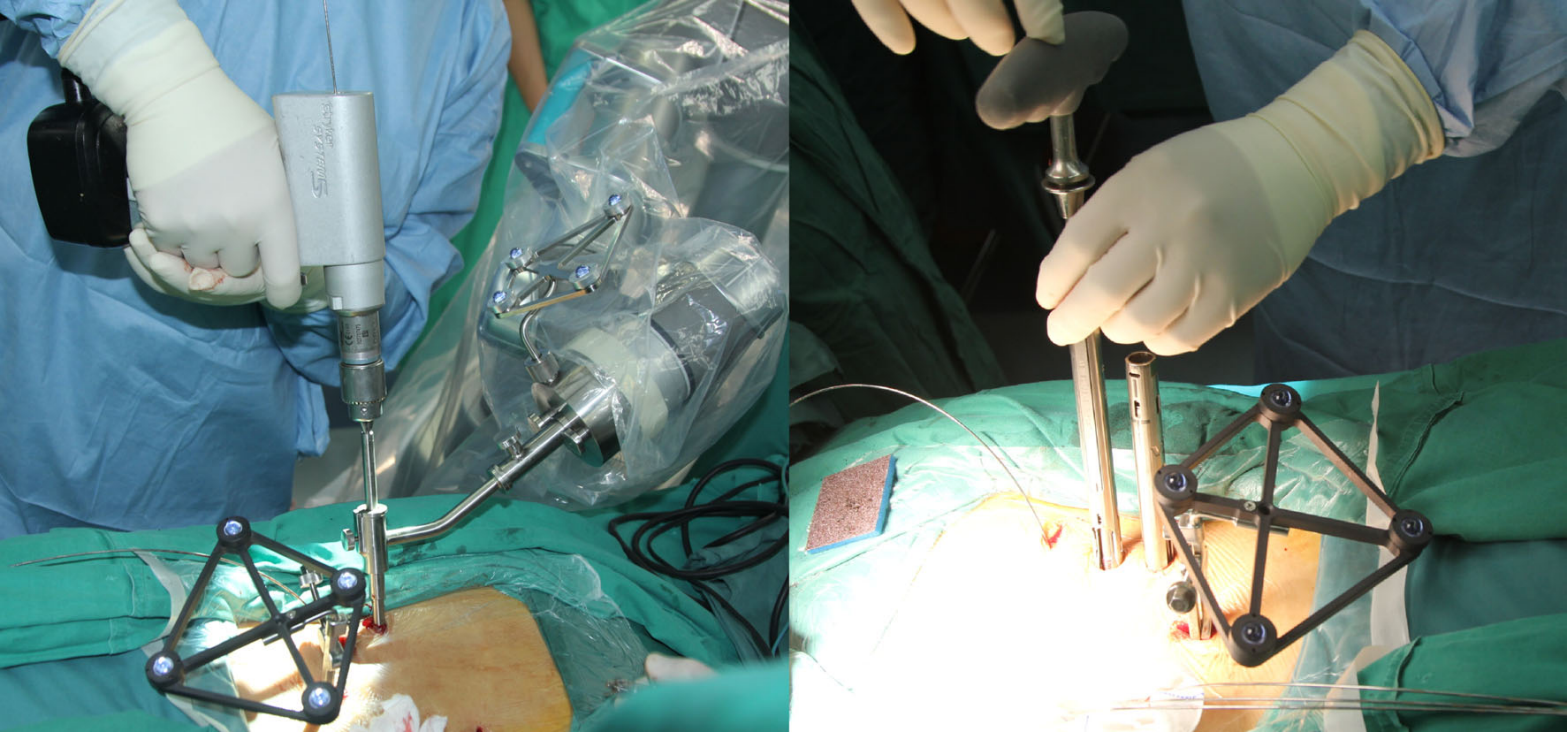
In the robot‐assisted group, K‐wires were inserted using the robot system, the pedicle was tapped, and the screws and rods were placed. Lateral and AP fluoroscopy were used to confirm the position of the K‐wire and/or screws.

**Figure 6:**
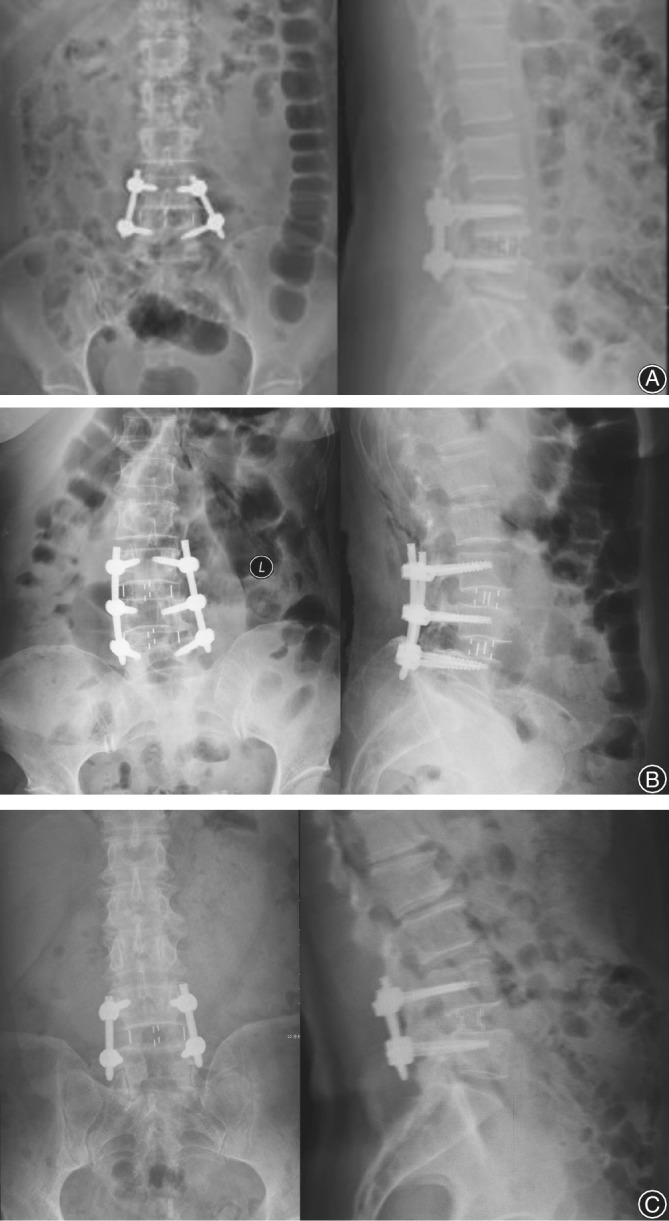
Anterioposterior and lateral X‐ray film after oblique lumbar interbody fusion (OLIF). (A) Female, 56 years old, with a diagnosis of lumbar spinal stenosis. (B) Female, 57 years old, with a diagnosis of degenerative spondylolisthesis. (C) Male, 61 years old, with a diagnosis of lumbar instability.

Next, patients were placed in the prone position. In the experimental group, a guide wire was introduced through a percutaneous incision on the back, and pedicle screws were inserted using a minimally invasive robot‐assisted technique (Fig. 5) Correct positioning of the screws was confirmed with fluoroscopy, and longitudinal connecting rods were installed (Fig. 6). In the control group, a posterior median approach was used. An incision was made through the skin and subcutaneous tissues, muscles were dissected, and pedicle screw entry points were exposed. Pedicle screws were inserted using a freehand technique with fluoroscopic guidance.

### *Outcomes*

#### *Accuracy of Pedicle Screw Placement*

Accuracy of pedicle screw placement was assessed on postoperative thin‐cut CT scans by a blinded, independent radiologist based on the modified Gertzbein and Robbins scale in which screws were graded as: A, without breach of the cortical layer of the vertebral body or pedicle; B, cortical breach of <2 mm; C, cortical breach of ≥2–<4 mm; D, cortical breach of ≥4–<6 mm; and E, cortical breach of ≥6 mm^8^. Pedicle screws that did not break the pedicle cortical layer in any direction (Group A) were recorded as accurately positioned pedicle screws. Pedicle screws graded as A were considered to have a perfect intrapedicular localization, while pedicle screws graded B–E had a poor trajectory^9^. All data were evaluated and recorded by a blinded, independent clinician.

#### *Duration of Surgery*

The duration of the entire surgery, the anterior procedure, and the posterior procedure were recorded. The duration of the entire surgery was defined as the time taken to perform all procedures after anesthesia, including positioning the patient, exposing the operative area, inserting the cage and screws, wound suture, and changing the patient's position between the anterior and posterior procedures. The duration of the anterior procedure was defined as the time taken to place the patient in the right lateral position, disinfect operation area, perform the anterior oblique approach and intervertebral fusion, and suture and dress the wound. The duration of the posterior procedure was defined as the time taken to place the patient in the prone position, disinfect operation area, perform posterior pedicle screw fixation, and suture and dress the wound.

#### *Intraoperative Blood Loss*

Intraoperative blood loss was measured as the volume of blood lost during surgery, calculated as the sum of the fluid in the suction bottle and the amount of blood in the gauze.

#### *Postoperative Drainage*

Postoperative drainage was measured as the volume of blood lost after surgery, calculated as the sum of the fluid in the drainage bottle.

#### *Lower Back and Leg Pain*

A visual analogue scale (VAS) was used to evaluate lower back and leg pain. Using a VAS ruler, the score was determined by measuring the distance (cm) on the 10‐cm line between the “no pain” anchor and the patient's mark, providing a range of scores from 0 to 10. A higher score indicated greater pain intensity. Patients described their lower back and leg pain intensity as 0 (no pain) to 10 (worst pain ever).

#### *Lumbar Function*

Lumbar function was evaluated before surgery, immediately after surgery, and at a 6‐month follow‐up using the ODI. The ODI is a principal condition‐specific outcome measure used in the management of spinal disorders and to assess patient progress in routine clinical practice. The ODI includes 10 sections: pain intensity, personal care, lifting, walking, sitting, standing, sleeping, sex life, social life, and traveling. Each section comprises six statements that are scored from 0 to 5. Intervening statements are scored according to rank. If more than one box is marked in each section, the highest score is used. If all10 sections are completed, the score is calculated as follows: total scored out of total possible score ×100. If one section is missed (or not applicable) the score is calculated: (total score/(5 × number of questions answered)) × 100%. Here, 0%–20% is considered mild dysfunction, 21%–40% is considered moderate dysfunction, 41%–60% is considered severe dysfunction, and 61%–80% is considered disability. For cases with a score of 81%–100%, patients are either long‐term bedridden or exaggerating the impact of pain on their life.

#### *Complication Rates*

Complication rates in the experimental or control groups were calculated as cases with complications/total cases.

### *Statistical Analysis*

Statistical analyses were conducted using SPSS 19.0 (IBM, USA). Continuous variables including age, mean operative time, intraoperative blood loss, postoperative drainage, and VAS and ODI scores, are reported as mean and standard deviation. Between‐group comparisons were made with the independent sample *t*‐test. Categorical variables, including number of patients, sex, and number of screws, are reported as a frequency or percentage, and between‐group comparisons were made with the χ^2^‐test. Significance was set at *P* < 0.05.

### *Ethical Approval*

This study was approved by the ethics committee of the Beijing Jishuitan Hospital, and all patients provided written informed consent.

## Results

### *Baseline Characteristics of Study Participants*

This study included 80 patients (31 men and 49 women) with a mean age of 63.80 years (range, 55 to 77 years). Among them, there were 34 cases of lumbar spondylolisthesis, 27 cases of lumbar spinal stenosis, 12 cases of lumbar intervertebral disc herniation, and 7 cases of lumbar instability. In the experimental group, 40 patients (16 men and 24 women), with a mean age of 63.45 years (range, 55–72 years), underwent percutaneous robot‐assisted minimally invasive pedicle screw insertion. In the control group, 40 patients (15 men and 25 women), with a mean age of 64.22 years (range, 55 to 77 years), underwent freehand fluoroscopy‐assisted pedicle screw insertion using a traditional open technique. There were no significant differences in baseline demographic characteristics between groups (Table 1).

**Table 1:** Patients baseline characteristics

Characteristic	Robot‐assisted	Freehand technique	*P*‐value
Number of patients	40	40	
Age at surgery (years)	63.45 ± 4.56	64.22 ± 6.19	0.525
Sex, number (%)			
Male	16 (40)	15 (37.5)	0.818
Female	24 (60)	25 (62.5)	
Diagnosis number (%)			
Lumbar spinal stenosis	19 (47.5)	21 (52.5)	0.577
Degenerative spondylolisthesis	18 (45)	14 (35)	
Lumbar instability	3 (7.5)	5 (12.5)	

### *Accuracy of Screw Placement*

A total of 344 pedicle screws were inserted in 80 patients;170 pedicle screws were inserted in the experimental group and 174 pedicle screws were inserted in the control group. With percutaneous robot‐assisted minimally invasive pedicle screw insertion, trajectories were Grade A in 167 screws, Grade B in 3 screws, and Grade C, D, or E in 0 screws. With freehand fluoroscopy‐assisted pedicle screw insertion using a traditional open technique, trajectories were Grade A in 162 screws, Grade B in 11 screws, Grade C in 1 screw, and Grade D or E in 0 screws. Accurate pedicle screw placement (Grade A) was significantly higher with percutaneous robot‐assisted minimally invasive pedicle screw insertion (98.2% [167/170]) compared to freehand fluoroscopy‐assisted pedicle screw insertion using a traditional open technique (93.1% [162/174]) (*P* < 0.05).

During surgery, one screw placed with the percutaneous robot‐assisted minimally invasive technique required intraoperative modification, possibly due to operator error and/or K‐wires slipping on the bone surface. Conversely, six pedicle screws required intraoperative modification during freehand fluoroscopy‐assisted pedicle screw insertion (Table 2).

**Table 2:** Clinical outcomes

Characteristic	Robot‐assisted	Freehand technique	*P*‐value
Number of screws	170	174	
Accuracy number (%)	167 (98.2%)	162 (93.1%)	0.039*
Inaccuracy number (%)	3 (1.8%)	12 (6.9%)	
Operation duration (min)	196.25 ± 62.85	230.63 ± 55.06	0.011*
Anterior operation duration (min)	103.88 ± 37.03	100.75 ± 28.57	0.674
Posterior operation duration (min)	77.13 ± 30.97	110.75 ± 27.00	0.000*
VAS of low back pain			
Preoperative	7.03 ± 1.25	7.28 ± 1.45	0.411
Postoperative	2.15 ± 1.15	3.35 ± 0.92	0.000*
6‐month follow‐up	1.20 ± 0.82	1.23 ± 0.66	0.881
VAS of leg pain			
Preoperative	7.23 ± 1.12	7.20 ± 1.62	0.936
Postoperative	2.33 ± 0.97	2.38 ± 1.06	0.826
6‐month follow‐up	1.05 ± 0.82	1.02 ± 0.80	0.890
ODI			
Preoperative	68.10 ± 6.72	68.25 ± 8.16	0.929
Postoperative	19.95 ± 5.33	21.15 ± 5.43	0.322
6‐month follow‐up	14.90 ± 5.18	15.25 ± 5.00	0.759
Intraoperative blood loss (mL)	165.00 ± 102.03	237.50 ± 167.47	0.022*
Intraoperative revision, no. of screws (%)	1 (0.01%)	6 (3.4%)	
Postoperative drainage (mL)	249.00 ± 102.73	0	0.000*
Surgical site infections	0	1	

*Statistically significant, *P* < 0.05.

VAS, visual analogue scale; ODI, oswestry disability index.

### *Duration of Surgery*

Mean operative time was significantly shorter for percutaneous robot‐assisted minimally invasive pedicle screw insertion (196.25 ± 62.85 min) compared to freehand fluoroscopy‐assisted pedicle screw insertion using a traditional open technique (230.63 ± 55.06 min) (*P* < 0.05). Mean operative time for the anterior procedure was not significantly different for robot‐assisted pedicle screw insertion (103.88 ± 37.03 min) compared to freehand fluoroscopy‐assisted pedicle screw insertion (100.75 ± 28.57 min) (*P* > 0.05). Mean operative time for the posterior procedure was significantly shorter for robot‐assisted pedicle screw insertion (77.13 ± 30.97 min) compared to freehand fluoroscopy‐assisted pedicle screw insertion (110.75 ± 27.00 min) (*P* < 0.05).

### *Clinical Outcomes*

The preoperative VAS score for lower back pain was not significantly different for patients undergoing percutaneous robot‐assisted minimally invasive pedicle screw insertion (7.03 ± 1.25) compared to those undergoing freehand fluoroscopy‐assisted pedicle screw insertion using a traditional open technique (7.28 ± 1.45). The immediate postoperative VAS score for lower back pain was significantly improved in patients undergoing robot‐assisted pedicle screw insertion (2.15 ± 1.15) compared to those undergoing freehand fluoroscopy‐assisted pedicle screw insertion (3.35 ± 0.92). The 6‐month follow‐up VAS score for lower back pain was not significantly different for patients undergoing robot‐assisted pedicle screw insertion (1.20 ± 0.82) compared to those undergoing freehand fluoroscopy‐assisted pedicle screw insertion (1.23 ± 0.66).

The preoperative, immediate postoperative, and 6‐month follow up VAS scores for leg pain were not significantly different for patients undergoing percutaneous robot‐assisted minimally invasive pedicle screw insertion (preoperative 7.23 ± 1.12; immediate postoperative 2.33 ± 0.97; 6‐month follow‐up 1.05 ± 0.82) compared to those undergoing freehand fluoroscopy‐assisted pedicle screw insertion using a traditional open technique (preoperative 7.20 ± 1.62; immediate postoperative 2.38 ± 1.06; 6‐month follow‐up 1.02 ± 0.80).

The preoperative, immediate postoperative, and 6‐month follow‐up ODI scores were not significantly different for patients undergoing percutaneous robot‐assisted minimally invasive pedicle screw insertion (preoperative 68.10 ± 6.72; immediate postoperative 19.95 ± 5.33; 6‐month follow‐up 14.90 ± 5.18) compared to those undergoing freehand fluoroscopy‐assisted pedicle screw insertion using a traditional open technique (preoperative 68.25 ± 8.16; immediate postoperative 21.15 ± 5.43; 6‐month follow‐up 15.25 ± 5.00).

### *Blood Loss*

Intraoperative blood loss was significantly less in patients undergoing percutaneous robot‐assisted minimally invasive pedicle screw insertion (165.00 ± 102.03 mL) compared to those undergoing freehand fluoroscopy‐assisted pedicle screw insertion using a traditional open technique (237.50 ± 167.47 mL) (*P* < 0.05). Postoperative drainage was 0 mL in patients undergoing robot‐assisted pedicle screw insertion and 249.00 ± 102.73 mL in those undergoing freehand fluoroscopy‐assisted pedicle screw insertion (Table 2).

### *Complications*

One patient undergoing freehand fluoroscopy‐assisted pedicle screw insertion using a traditional open technique experienced an infection at the surgical site. One patient undergoing percutaneous robot‐assisted minimally invasive pedicle screw insertion experienced weakness in the left hip flexor. Two patients undergoing freehand fluoroscopy‐assisted pedicle screw insertion using a traditional open technique experienced complications. One patient experienced weakness in the left hip flexor and the other had delayed wound healing. All complications were resolved at 1 month postoperatively.

## Discussion

The present study compared the effects of percutaneous robot‐assisted minimally invasive pedicle screw insertion with freehand fluoroscopy‐assisted pedicle screw insertion using a traditional open technique on clinical and functional outcomes in elderly patients undergoing an OLIF procedure. The findings showed that 98.2% of pedicle screws had a perfect intrapedicular localization following percutaneous robot‐assisted minimally invasive pedicle screw insertion, compared to 91.6% for freehand fluoroscopy‐assisted pedicle screw insertion using a traditional open technique, confirming that the accuracy of robot‐assisted pedicle screw placement technology exceeds that of the human freehand technique.

Precisely determining the location and orientation of pedicle screw placement is challenging^10^, ^11^, ^12^. Traditionally, surgeons rely on preoperative imaging (X‐ray, CT, MRI), 3D printed models, and are assisted by intraoperative X‐ray fluoroscopy^13^, ^14^. However, freehand operations can be limited by a surgeon's skill level, experience, and fatigue^15^. Robot‐assisted pedicle screw insertion has a targeted accuracy of ±1 mm, and minimally invasive, percutaneous screw placement can limit the influence of soft tissue tension on screw trajectory^16^, ^17^. These data, together with the findings from the current study, suggest that percutaneous robot‐assisted minimally invasive pedicle screw insertion could play an important role in accurately controlling the position and direction of pedicle screw placement, showing obvious benefits when used in posterior internal fixation in OLIF.

Mean operative time was significantly shorter for percutaneous robot‐assisted minimally invasive pedicle screw insertion compared to freehand fluoroscopy‐assisted pedicle screw insertion using a traditional open technique. There was no significant difference in mean duration of the anterior procedure with robot‐assisted pedicle screw insertion compared to freehand fluoroscopy‐assisted pedicle screw insertion. This was not surprising as both techniques require insertion of an intervertebral fusion cage. However, mean operative time for the posterior procedure was 33.62 min shorter for robot‐assisted pedicle screw insertion compared to freehand fluoroscopy‐assisted pedicle screw insertion. Robot‐assisted pedicle screw insertion requires small surgical incisions, causes minimal trauma to soft tissue, and achieves a high accuracy of screw placement. In contrast, freehand fluoroscopy‐assisted pedicle screw insertion requires a large incision, extensive exposure of soft tissue, and suture and dressing of the wound, and can involve repeated fluoroscopy and adjustment of the screw position. These data suggest that robot‐assisted minimally invasive, percutaneous pedicle screw insertion in OLIF is more time‐effective than a freehand technique.

Visual analogue scale scores for lower back and leg pain were substantially improved in the postoperative period compared to before surgery in patients undergoing percutaneous robot‐assisted minimally invasive pedicle screw insertion and in those undergoing freehand fluoroscopy‐assisted pedicle screw insertion using a traditional open technique, suggesting that patients in this study were suffering from nerve compression preoperatively. There were no significant differences in VAS scores for lower back pain before surgery or at the 6‐month follow‐up, or in VAS scores for leg pain before surgery, in the immediate postoperative period, and at the 6‐month follow up in patients undergoing robot‐assisted pedicle screw insertion compared to freehand fluoroscopy‐assisted pedicle screw insertion. In the immediate postoperative period, robot‐assisted pedicle screw insertion was associated with significantly less lower back pain and patients were more mobile, likely because their procedure required a smaller incision. At the 6‐month follow up, all incisions had healed, and VAS scores in patients undergoing robot‐assisted pedicle screw insertion and freehand fluoroscopy‐assisted pedicle screw insertion were similar. These findings confirm that OLIF is effective for the treatment of lumbar degenerative diseases with nerve compression^18^, ^19^.

Lumbar function, as evidenced by ODI scores, was substantially improved in the postoperative period compared to before surgery in patients undergoing percutaneous robot‐assisted minimally invasive pedicle screw insertion and in those undergoing freehand fluoroscopy‐assisted pedicle screw insertion using a traditional open technique. There were no significant differences in lumbar function before surgery, in the immediate postoperative period, and at the 6‐month follow up in patients undergoing robot‐assisted pedicle screw insertion compared to those undergoing freehand fluoroscopy‐assisted pedicle screw insertion. This was unexpected as pain after surgery was more severe in patients that underwent freehand fluoroscopy‐assisted pedicle screw insertion, but the finding may have been the result of postoperative analgesia.

Intraoperative blood loss and postoperative drainage were significantly less in patients undergoing percutaneous robot‐assisted minimally invasive pedicle screw insertion compared to those undergoing freehand fluoroscopy‐assisted pedicle screw insertion using a traditional open technique. Robot‐assisted pedicle screw insertion is associated with less soft‐tissue damage. The surgeon can more precisely predict the trajectory of the screw such that the placement of each screw requires a 1‐cm skin incision, followed by blunt separation of fascia and muscle, with minimal damage to soft tissues and blood vessels. As the wound and soft tissue damage are minimal during robot‐assisted pedicle screw insertion, there is no postoperative bleeding; therefore, postoperative drainage tubes are not needed. Freehand fluoroscopy‐assisted pedicle screw insertion requires a 10‐cm skin incision, separation of a broad range of soft tissues, including muscle, and exposure of the bilateral lamina to the lateral side of the facet joint. More surgical procedures are needed, which leads to longer operative times and increased intraoperative blood loss. Postoperative drainage is substantial, which could result in blood loss and have adverse effects on hemodynamic stability.

This study was associated with several limitations. First, there is a paucity of data linking accuracy of screw placement to clinical outcomes. Further large size studies with a longer follow up are required to confirm the clinical relevance of our findings. Second, subgroup analyses exploring the effect of patients’ baseline demographic and clinical variables on our outcomes were not performed due to small sample sizes. These data are important to inform patient selection and will be a topic of future research.

### *Conclusion*

Percutaneous robot‐assisted minimally invasive pedicle screw insertion was more accurate than freehand fluoroscopy‐assisted pedicle screw insertion using a traditional open technique in elderly patients with lumbar degenerative diseases undergoing OLIF. Robot‐assisted pedicle screw insertion in OLIF is time effective, ensures internal fixation stability, reduces surgical trauma, accelerates early postoperative recovery, and decreases the risk of surgical complications.

## References

[1] Hayashi D , Roemer FW , Mian A , Gharaibeh M , Muller B , Guermazi A . Imaging features of postoperative complications after spinal surgery and instrumentation. AJR Am J Roentgenol, 2012, 199: W123–W129. 22733920 10.2214/AJR.11.6497

[2] Borcek AO , Suner HI , Emmez H , Kaymaz M , Aykol S , Pasaoglu A . Accuracy of pedicle screw placement in thoracolumbar spine with conventional open technique. Turk Neurosurg, 2014, 24: 398–402. 24848181 10.5137/1019-5149.JTN.10445-14.1

[3] Kaneyama S , Sugawara T , Sumi M . Safe and accurate midcervical pedicle screw insertion procedure with the patient‐specific screw guide template system. Spine, 2015, 40: E341–E348. 25584951 10.1097/BRS.0000000000000772

[4] Larson AN , Polly DW Jr , Guidera KJ , *et al* The accuracy of navigation and 3D image‐guided placement for the placement of pedicle screws in congenital spine deformity. J Pediatr Orthop, 2012, 32: e23–e29. 22892631 10.1097/BPO.0b013e318263a39e

[5] Silvestre C , Mac‐Thiong JM , Hilmi R , Roussouly P . Complications and morbidities of mini‐open anterior retroperitoneal lumbar interbody fusion: oblique lumbar interbody fusion in 179 patients. Asian Spine J, 2012, 6: 89–97. 22708012 10.4184/asj.2012.6.2.89 PMC3372554

[6] Phan K , Mobbs RJ . Oblique lumbar interbody fusion for revision of non‐union following prior posterior surgery: a case report. Orthop Surg, 2015, 7: 364–367. 26791588 10.1111/os.12204 PMC6583715

[7] Mehren C , Korge A . Minimally invasive anterior oblique lumbar interbody fusion (OLIF). Eur Spine J, 2016, 25: 471–472. 26914094 10.1007/s00586-016-4465-9

[8] Gertzbein SD , Robbins SE . Accuracy of pedicular screw placement in vivo. Spine, 1990, 15: 11–14. 2326693 10.1097/00007632-199001000-00004

[9] Aoude AA , Fortin M , Figueiredo R , Jarzem P , Ouellet J , Weber MH . Methods to determine pedicle screw placement accuracy in spine surgery: a systematic review. Eur Spine J, 2015, 24: 990–1004. 25749690 10.1007/s00586-015-3853-x

[10] Yoshida G , Kanemura T , Ishikawa Y . Percutaneous pedicle screw fixation of a Hangman's fracture using intraoperative, full rotation, three‐dimensional image (O‐arm)‐based navigation: a technical case report. Asian Spine J, 2012, 6: 194–198. 22977699 10.4184/asj.2012.6.3.194 PMC3429610

[11] Verma R , Krishan S , Haendlmayer K , Mohsen A . Functional outcome of computer‐assisted spinal pedicle screw placement: a systematic review and meta‐analysis of 23 studies including 5,992 pedicle screws. Eur Spine J, 2010, 19: 370–375. 20052504 10.1007/s00586-009-1258-4 PMC2899753

[12] Tian W , Fan MX , Liu YJ . Robot‐assisted percutaneous pedicle screw placement using three‐dimensional fluoroscopy: a preliminary clinical study. Chin Med J, 2017, 130: 1617–1618. 28639580 10.4103/0366-6999.208251 PMC5494928

[13] Carbone JJ , Tortolani PJ , Quartararo LG . Fluoroscopically assisted pedicle screw fixation for thoracic and thoracolumbar injuries: technique and short‐term complications. Spine, 2003, 28: 91–97. 12544964 10.1097/00007632-200301010-00021

[14] Assaker R , Reyns N , Vinchon M , Demondion X , Louis E . Transpedicular screw placement: image‐guided versus lateral‐view fluoroscopy: in vitro simulation. Spine, 2001, 26: 2160–2164. 11698897 10.1097/00007632-200110010-00024

[15] Chan A , Parent E , Narvacan K , San C , Lou E . Intraoperative image guidance compared with free‐hand methods in adolescent idiopathic scoliosis posterior spinal surgery: a systematic review on screw‐related complications and breach rates. Spine J, 2017, 17: 1215–1229. 28428081 10.1016/j.spinee.2017.04.001

[16] Kim HJ , Lee SH , Chang BS , *et al* Monitoring the quality of robot‐assisted pedicle screw fixation in the lumbar spine by using a cumulative summation test. Spine, 2015, 40: 87–94. 25575085 10.1097/BRS.0000000000000680

[17] van Dijk JD , van den Ende RP , Stramigioli S , Kochling M , Hoss N . Clinical pedicle screw accuracy and deviation from planning in robot‐guided spine surgery: robot‐guided pedicle screw accuracy. Spine, 2015, 40: E986–E991. 25943084 10.1097/BRS.0000000000000960

[18] Fujibayashi S , Hynes RA , Otsuki B , Kimura H , Takemoto M , Matsuda S . Effect of indirect neural decompression through oblique lateral interbody fusion for degenerative lumbar disease. Spine, 2015, 40: E175–E182. 25394317 10.1097/BRS.0000000000000703

[19] Ohtori S , Orita S , Yamauchi K , *et al* Change of lumbar ligamentum flavum after indirect decompression using anterior lumbar interbody fusion. Asian Spine J, 2017, 11: 105–112. 28243378 10.4184/asj.2017.11.1.105 PMC5326718

